# Structural and Biochemical Characterization of the Diaminopimelate Epimerase DapF From *Pseudomonas aeruginosa* PA14

**DOI:** 10.1002/prot.70161

**Published:** 2026-07-28

**Authors:** Philipp Schneider, Claudia Hanko, Konrad Büssow, Stefan Schmelz, Wulf Blankenfeldt

**Affiliations:** ^1^ Department Structure and Function of Proteins Helmholtz Centre for Infection Research Braunschweig Germany; ^2^ Institute for Biochemistry, Biotechnology and Bioinformatics Technische Universität Braunschweig Braunschweig Germany

**Keywords:** diaminopimelate epimerase, lysine biosynthesis, peptidoglycan biosynthesis, *Pseudomonas aeruginosa*, RppH, X‐ray structure

## Abstract

The emergence of antibiotic resistance in bacteria is a major threat to global public health. To combat increasingly drug‐resistant bacteria like *Pseudomonas aeruginosa* , new targets for pharmaceuticals need to be explored. One such potential target for *P. aeruginosa* infections is the diaminopimelate epimerase DapF, a protein that plays an essential role in bacterial lysine and peptidoglycan synthesis. In this study, the diaminopimelate epimerase of *Pseudomonas aeruginosa* PA14 (PA14_69690) is characterized by a crystal structure at 1.69 Å resolution, revealing a homodimer in the open conformation in which one of the monomers was found inactivated by disulfide formation between the two catalytic cysteines of the active site. Further, the influence of substrate and product on the stability of the enzyme has been investigated, finding that both significantly increase the melting temperature. We determined enzyme kinetic parameters with a coupled enzyme assay. With *K*_*M*_ = 211 μM (95% confidence interval: 169–267 μM) and *k*_cat_ of 76 s^−1^ (95% confidence interval: 69–85 s^−1^), *P. aeruginosa* PA14 DapF has activity levels comparable to homologues from other bacterial species. Finally, we show that a previously observed interaction between DapF and the Nudix hydrolase RppH in *E. coli* is conserved in *P. aeruginosa* . Soluble recombinant *P. aeruginosa* RppH could only be obtained when the hydrolase was co‐expressed with DapF, hinting at a moonlighting chaperone activity of *P. aeruginosa* DapF. The catalytic activity of DapF was not affected by complex formation with RppH.

## 1 Introduction

The introduction of Ehrlich's Salvarsan [1], Domagk's sulfonamide [2] and Fleming's penicillin [3] were significant advances in the treatment of bacterial infections. After these first antibiotics, researchers discovered and developed more and more compounds that enabled reliable treatment of many bacterial diseases [4]. However, due to their extensive use and sometimes abuse in patients as well as their overuse in livestock farming [5, 6, 7], the golden age of antibiotics has meanwhile come to an end, since bacteria began to acquire resistance genes against these pharmaceuticals [8]. Today the WHO declares antibiotic resistance to be one of the major threats to global health [9, 10], and so‐called “superbugs”, bacteria possessing resistances against many classes of available antibiotics, have become a large concern. One of these superbugs is *Pseudomonas aeruginosa* , a Gram‐negative, opportunistic bacterium that infects a wide range of organisms [8] as well as many human tissues [11, 12]. Individuals with a compromised immune system or patients with pulmonary diseases such as cystic fibrosis are at particularly high risk for chronic infections with this pathogen [13]. Its repertoire of resistance mechanisms confers protection against almost all antibiotics on the market and makes the treatment of infections very difficult [8, 14, 15]. This urges researchers to find new targets in *P. aeruginosa* that are eligible for the development of new antibacterial compounds. A putative target is diaminopimelate epimerase DapF, which acts as a pyridoxal‐5′‐phosphate independent epimerase and also moonlights as an activator of bacterial mRNA degradation (Figure 1A). In its enzymatic function, it catalyzes the stereoconversion of L,L‐diaminopimelate (L,L‐DAP) to D,L‐diaminopimelate (*meso*‐diaminopimelate; D,L‐DAP), a compound involved in two essential metabolic pathways in bacteria, namely peptidoglycan and lysine biosynthesis [16, 17]. The peptidoglycan sacculus is essential for most bacteria to resist osmotic pressure and to anchor surface proteins. Here, D,L‐DAP is an important cross‐linking building block [18]. Lysine, on the other hand, is one of the 20 proteinogenic amino acids that are required for the biosynthesis of proteins, playing a crucial role in almost all biological processes [19]. The catalytic and selectivity mechanisms of the homodimeric DapF enzymes from several bacterial species have been thoroughly investigated and explain how the enzyme generally activates two cysteine residues to perform the epimerization reaction (Figure 1B, reviewed by Fischer et al. [20]) while it at the same time selects for the L‐conformation of the distal, non‐converted α‐amino group, thereby ensuring that D,L‐DAP does not get converted into the D,D‐product [21, 22]. Interestingly, Lamer and colleagues have recently identified DapF enzymes that utilize two catalytic serines instead of cysteines, while preserving the same overall fold [23]. Replacing these serines for cysteines in the enzyme from *Anabaena* sp. YBS01 led to inactivation, showing that the DapF enzymes have evolved to fine‐tune the pKa values of their catalytic residues.

**FIGURE 1:**
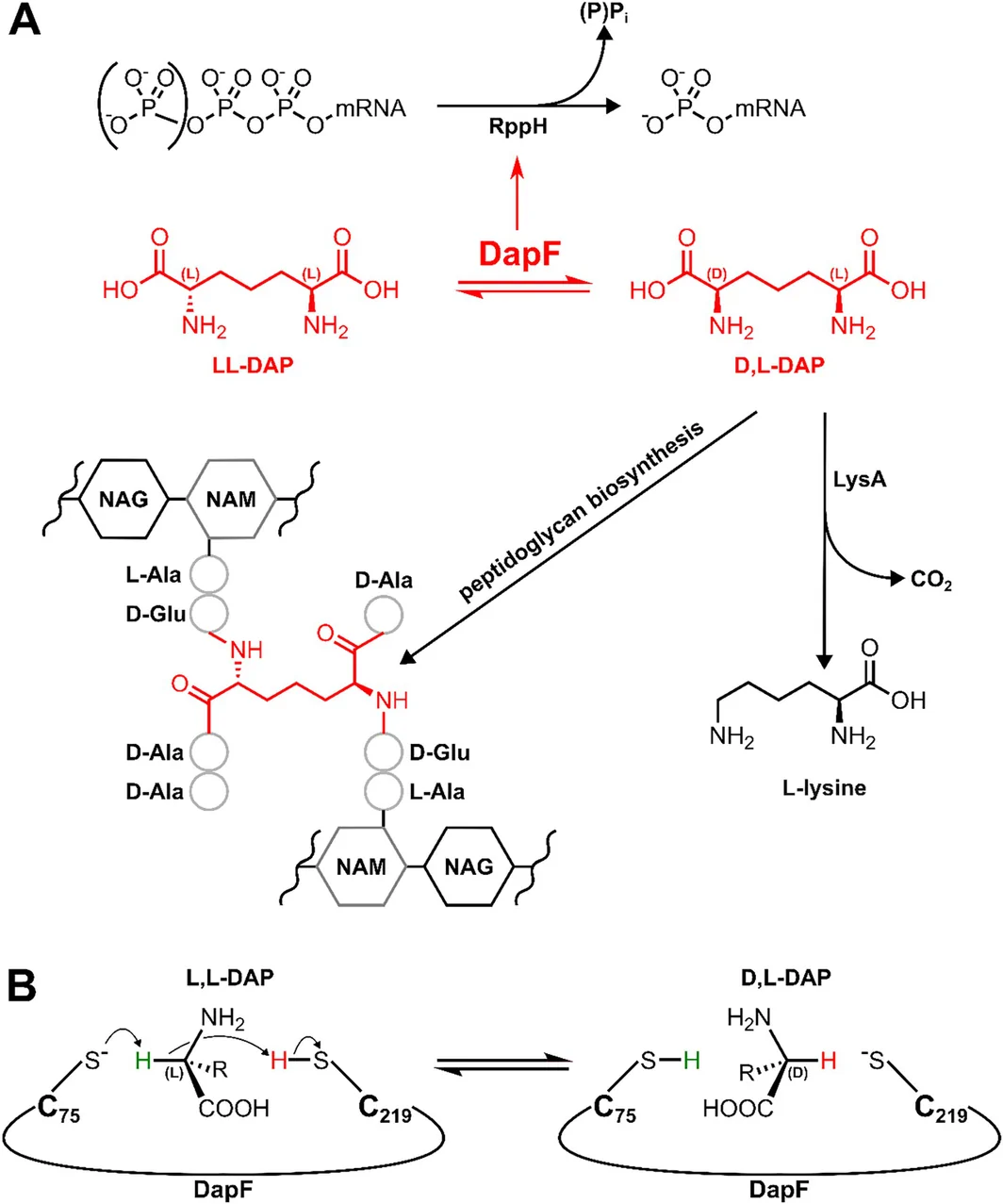
Activities of DapF. (A) DapF catalyzes the epimerization of L,L‐diaminopimelate (L,L‐DAP) to D,L‐diaminopimelate (D,L‐DAP) (red), which can then be incorporated into the peptidoglycan layer of bacteria or further be decarboxylated to L‐lysine through the action of LysA. In *E. coli* , DapF has also been shown to engage in a complex with RppH and activate this Nudix hydrolase to accelerate the rate‐limiting 5′‐de(pyro)phosphorylation step in mRNA degradation. (B) The catalytic activity of DapF is routed in two cysteine or serine residues (C75 and C219 in *P. aeruginosa* PA14 DapF) that de‐ and reprotonate one of the proximal chiral centers of diaminopimelate. NAG, N‐acetylglucosamine; NAM, N‐acetylmuramic acid.

Another unexpected finding is that DapF engages in a complex and thereby activates RppH (also annotated as NudH or YgdP), a Nudix hydrolase that catalyzes the rate‐limiting 5′‐de(pyro)phosphorylation step in mRNA degradation, hinting at a physiological role beyond its epimerase activity [24, 25, 26]. However, this interaction has until now only been demonstrated in *E. coli* , and it is not clear if it is preserved in other bacterial species as well.

Because of its catalytic activity, DapF is essential or predicted to be essential for most bacteria [27], and the moonlighting role in mRNA degradation may add a second layer to its importance in bacterial physiology. In addition, the absence of this enzyme in humans makes it an interesting target for new antimicrobial compounds [28], and several studies discuss or report strategies to inhibit DapF, including breaking DapF‐homodimer formation [21, 29, 30, 31, 32].

Here, we characterize the DapF protein of the nosocomial pathogen *P. aeruginosa* PA14 at the structural and enzyme‐kinetic level. We also confirm its interaction with *P. aeruginosa* RppH, suggesting that its role in RNA homeostasis is conserved in this opportunistic pathogen.

## 2 Materials and Methods

### 2.1 Protein Expression and Purification

DapF (PA14_69690) and the diaminopimelate decarboxylase LysA (PA14_69670) from *Pseudomonas aeruginosa* PA14 were overexpressed with a Tobacco Etch Virus (TEV) protease‐cleavable N‐terminal Strep‐tagII in *E. coli* BL21(DE3). Co‐expression of DapF and RppH was achieved with a pET vector with two expression cassettes encoding for DapF with N‐terminal His_6_‐tag and RppH (PA14_04390) with N‐terminal Strep‐tagII. For overexpression, ZYM‐5052 autoinduction media was supplemented with 100 μg/mL kanamycin. The cultures were shaken for 2.5 h with 130 rpm at 37°C. Subsequently, the temperature was decreased to 20°C before shaking overnight. Cells were then harvested and for the purification of DapF or LysA the pellet was resuspended in washing buffer containing 100 mM Tris/HCl pH 8, 150 mM NaCl, 1 mM DTT and a complete protease inhibitor cocktail tablet (Roche). The cells were lysed by sonication (30 min, 59% amplitude, 1 s on, 9 s off; Bandelin Sonoplus GM2200.2) and the lysate was subsequently centrifuged with 37 000 × *g* for 30 min. Supernatant was filtered with a 0.45 μm cut‐off filter and loaded on a Strep‐Tactin column (IBA Lifesience/Cytiva). The column was washed with washing buffer until a stable UV signal was obtained. Bound protein of interest was eluted with washing buffer supplemented with 5 mM desthiobiotin. Elution fractions containing the enzyme were united, concentrated and supplemented with TEV protease to remove the Strep‐tagII. After overnight incubation, the solution was again passed over a Strep‐Tactin column (IBA Lifesience/Cytiva) and a 5 mL His‐Trap column (Cytiva) to remove uncleaved DapF enzyme and the His‐tagged TEV protease. The flow‐through was collected and loaded onto a Superdex 200 26/600 column (Cytiva), developed in washing buffer. For LysA the Strep‐tagII was not removed and the affinity‐purified protein was directly loaded on the Superdex 200 26/600 column. Fractions containing protein were collected. DapF (*M*_*w*_ = 30 464, *ε* = 26 470 M^−1^ cm^−1^) and LysA (*M*_*w*_ = 48 171, *ε* = 34 840 M^−1^ cm^−1^) were concentrated to 5.1 and 39.5 mg/mL, respectively, by ultrafiltration, before being aliquoted and stored at −80°C until further use.

The DapF/RppH complex was purified in a similar manner on a Strep‐Tactin column with buffer A (50 mM HEPES pH 8.0, 300 mM NaCl, 1 mM DTT, 5% glycerol) and elution buffer B (5 mM desthiobiotin in buffer A). Elution peak fractions were pooled and a protein concentration of 5.1 g/L was measured by UV, using *M*_*w*_ = 53 699 and *ε* = 80 900 M^−1^ cm^−1^ (calculated for the DapF/RppH heterodimeric unit, which constitutes half of the heterotetramer). On the same day, protein aliquots were used for SEC‐MALS or stored at −80°C until further use. The His‐ and Strep‐tagII were not removed.

### 2.2 SEC‐MALS (Size Exclusion Chromatography Coupled With Multi Angle Light Scattering)

The molecular weight of DapF and the DapF/RppH complex in solution were analyzed with size‐exclusion chromatography (SEC) coupled to a multi angle light scattering (MALS) device. Measurements were carried out at room temperature with an Agilent 1260 HPLC (Agilent Technologies) coupled to MALS (mini Dawn TREOS II/Wyatt) and a differential refractive index (dRI, Optilab/Wyatt) detectors. 260 μg DapF were injected on a pre‐equilibrated Superdex 200 10/300 (Cytiva). The running buffer contained 100 mM Tris/HCl pH 8 and 150 mM NaCl. For the DapF/RppH complex, the buffer composition was 50 mM HEPES/NaOH pH 8, 300 mM NaCl, 1 mM DTT and 5% (v/v) glycerol, and 180 μg of protein were applied. Data were recorded and analyzed in ASTRA version 8.2.2 (Wyatt).

### 2.3 Thermal Stability

Protein storage buffer was exchanged against 100 mM phosphate buffer pH 8 using Zeba spin desalting columns from Thermo Fisher Scientific Inc. according to the manufacturer's protocol. Thermal stability of 0.5 μM enzyme supplemented with 20 mM of the compounds L,L‐DAP, D,L‐DAP and racemic 2‐aminoadipic acid and the enzyme alone was examined by nanoscale differential scanning fluorimetry (nanoDSF). Here, the changes in the intrinsic fluorescence of tyrosine and tryptophan residues were monitored using a Prometheus Panta system from NanoTemper Technologies. The fluorescence ratio *F*_350_/*F*_330_ was plotted against the temperature, and melting points were identified by calculating the inflection points with the smoothed first derivative. The first derivatives were normalized and plotted in a graph with GraphPad Prism 8.4.3. The temperature range covered in the measurement was 15°C–95°C with a heat ramp of 7°C/min.

### 2.4 Enzyme Kinetic Assay

The reaction of L,L‐DAP to D,L‐DAP, catalyzed by DapF, was measured with a coupled enzyme assay [16]. Purified LysA converted D,L‐DAP to lysine (Figure 1). Oxidative deamination of lysine by atmospheric oxygen, catalyzed by lysine oxidase from *Trichoderma viride* (Sigma‐Aldrich L6150), resulted in formation of H_2_O_2_ (1:1 stoichiometry), which was used by horseradish peroxidase (HRP, Sigma‐Aldrich P6782) to oxidize ABTS (2,2′‐azino‐bis[3‐ethylbenzothiazoline‐6‐sulfonic acid] diammonium salt) to the stable, colored ABTS^•+^ radical. The latter reaction has a stoichiometry of 2 mol of ABTS^•+^ generated per 1 mol of H_2_O_2_ [33]. The oxidation of ABTS was measured at 412 nm using the extinction coefficient of *ε* = 32 400 M^−1^ cm^−1^ from Kumar et al. [33].

Reactions were performed in a final volume of 40 μL in 100 mM potassium phosphate buffer (pH 8.0) in a transparent polystyrene 384‐well plate (Greiner 781101). Each reaction contained 0.1 U/mL lysine oxidase (25 U/mg), 1 U/mL (1 μg/mL) HRP, 14.4 mM ABTS, 15.6–500 μM L,L‐DAP, 8.6 μM LysA and 4 nM DapF or 4 nM DapF/RppH complex. Absorbance at 412 nm (bandwidth 3.5 nm) was measured every 15 s in a Tecan Spark plate reader at room temperature (22°C).

To set up the reactions, four solutions at fourfold final concentration were prepared in the phosphate buffer in polypropylene tubes: 4 × oxi‐mix (lysine oxidase, HRP, ABTS), 4 × L,L‐DAP, 4 × LysA and 4 × DapF (diluted from a 1 mg/mL stock). Each microtiter plate well received 10 μL each of 4 × oxi‐mix, 4 × L,L‐dap and 4 × LysA. The wells were mixed by shaking the plate briefly at 2000 rpm on an Eppendorf Thermomixer C and A_412_ was measured for ~2 min, until a stable absorbance was obtained. Empty wells were filled with 4 × DapF and 10 μL volumes were dispensed from these wells to the previously pipetted 30 μL reaction mixes with a multichannel pipette (minimizing contact time of the enzyme with the polystyrene surface). The plate was shaken again and A_412_ was measured every 15 s for 5 min. A path length of *d* = 0.33 cm was determined by measuring a 40 μL reaction mix without ABTS with the plate reader's path length correction function. The initial velocities of *A*_412_ increase over time were determined (Δ*A*_412_/Δ*t*) with GraphPad Prism using *A*_412_ values between 0.5 to 1.5 min reaction time. Reaction rates *v* were calculated, considering the 2:1 stoichiometry, with *v* = (Δ*A*_412_/Δ*t*)/(*d*·*ε*·2). Data were fitted to the Michaelis–Menten equation with GraphPad Prism to determine values for *V*_max_, *K*_*M*_ and *k*_cat_ = *V*_max_/(4 nM) together with 95% confidence intervals (CI) for these values.

### 2.5 Protein Crystallization

DapF was crystallized by the sitting drop vapor diffusion method at room temperature. With a Crystal Gryphon dispensing robot (Art Robbins Instruments), 10 mg/mL DapF were set up with the index crystallization screen from Hampton Research. Each drop contained 200 nL DapF protein solution and 200 nL mother liquor. First crystals were detected 36 h after set up with precipitant containing 0.49 M NaH_2_PO_4_ and 0.91 M K_2_HPO_4_. Crystals were harvested and cryoprotected with 10% (v/v) (2R,3R)‐2,3‐butanediol in the crystallization solution before mounting in a nylon loop and flash‐cooling in liquid nitrogen.

### 2.6 Diffraction Data Collection

Diffraction data were collected at 100 K on beamline P11 of the PETRAIII synchrotron of the Deutsches Elektronensynchrotron (DESY Hamburg, Germany) [34, 35]. 3600 frames were recorded over a total rotation range of 360° with an exposure time of 40 ms per image. The distance to the PILATUS 6MF‐0109 detector was 298.5 mm and the wavelength was set to 1.033 Å, corresponding to 12 keV. Recorded data were processed using the autoPROC toolbox (Global Phasing) [36] executing XDS [37], pointless [38] and aimless [39].

### 2.7 Structure Solution and Refinement

Initial phases were obtained by molecular replacement with the BALBES [40] online server, which identified DapF from *Haemophilus influenzae* (PDB code: 2GKE) [21] as the most suitable search model (54% sequence identity). Model building subsequently involved automated model building [41] and maximum likelihood refinement [42] in the phenix software suite [43], iterated with manual rebuilding cycles in Coot [44]. PyMOL [45] was used to render representations of the structure. The structure has been deposited in the Protein Data Bank PDB [46] with entry ID 8QZY.

## 3 Results and Discussion

### 3.1 Biochemical and Biophysical Characterization of *P. aeruginosa* PA14 DapF

Proteins related to DapF, including other epimerases and enzymes with different catalytic activity such as dehydratases or phenazine biosynthesis protein PhzF [47], are generally found as homodimers, and for *E. coli* and *H. influenzae* DapF dimer formation has been analyzed in detail by Hor et al. [31]. To corroborate the dimeric state of *P. aeruginosa* PA14 DapF in solution, we performed a SEC‐MALS experiment (Figure 2A). The calculated molecular weight underlying the single peak was 58.4 kDa, slightly less than the theoretical mass for a homodimer of 60.6 kDa calculated using the ExPASy server [48], showing that the enzyme indeed is a homodimer.

**FIGURE 2:**
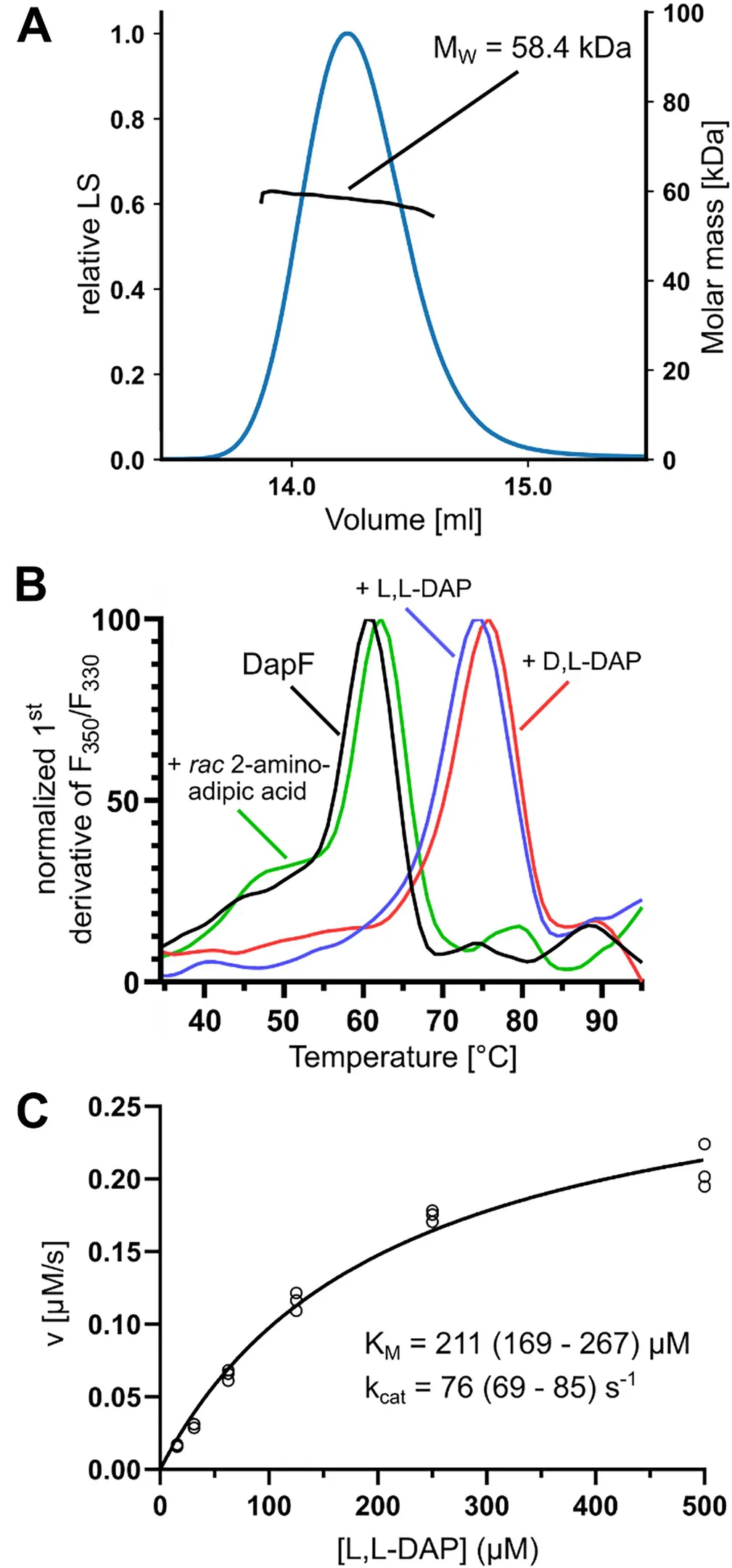
Biophysical and biochemical characterization of *P. aeruginosa* PA14 DapF. (A) Chromatogram of the SEC‐MALS analysis. The *x*‐axis shows the retention volume of the SEC column. Relative light scattering is shown by the blue curve. The black line refers to the right *y*‐axis and shows the calculated molecular weight. (B) Thermal stability profiles. The 1st derivatives of the melting curves show the contribution of L,L‐DAP (blue), D,L‐DAP (red), racemic 2‐aminoadipic acid (green) to the thermal stability compared to apo DapF (black). (C) Determination of enzyme kinetic parameters of *P. aeruginosa* PA14 DapF. Initial velocities plotted against the corresponding substrate concentration. Values in parentheses represent the 95% confidence interval obtained from nonlinear regression of three technical replicates per substrate concentration.

The gene encoding the enzyme analyzed here (PA14_69690) is neighboured to a diaminopimelate decarboxylase LysA (PA14_69670), suggesting that it indeed produces a DapF diaminopimelate epimerase that should bind the substrate L,L‐DAP and the product D,L‐DAP. We therefore tested if these compounds as well as the diaminopimelate analogue 2‐aminoadipic acid stabilize the enzyme against thermal denaturation. Indeed, L,L‐DAP and D,L‐DAP increased the melting temperature significantly and to a similar extent (L,L‐DAP: 13.5 K; D,L‐DAP: 15 K). Racemic 2‐aminoadipic acid, on the other hand, had only minute effects on the stability of PA14 DapF (ca. 1.5 K), suggesting that it is not accepted by DapF and indicating that the distal, non‐converted α‐aminocarboxylate group of DAP is essential for substrate recognition (Figure 2B).

Finally, we measured enzyme kinetic parameters for *P. aeruginosa* PA14 DapF, using a modified coupled enzyme assay [16]. To ensure the reaction is dominated by DapF, the concentration of all other enzymes was increased in a control experiment without observing any effects. Nonlinear regression using the Michaelis–Menten equation yielded *K*_*M*_ = 211 μM (95% CI: 169–267 μM) and *V*_max_ = 0.303 μM s^−1^ (95% CI: 0.274–0.339 μM s^−1^), corresponding to a *k*_cat_ of 76 s^−1^ (95% CI: 69–85 s^−1^) (Figure 2C). This puts *P. aeruginosa* PA14 DapF at par with other previously characterized diaminopimelate epimerases (Table S1).

### 3.2 Structure of *P. aeruginosa* PA14 DapF

*P. aeruginosa* PA14 DapF crystallized in space group P 2_1_ 2_1_ 2_1_, containing two monomers in the asymmetric unit. Crystals diffracted to 1.69 Å resolution (Table 1) and the structure was solved by molecular replacement, yielding a final model that contains 276 and 278 residues (out of 278 possible) in monomer A and B, respectively, with final *R*‐values of *R*_work_ = 17.7% and *R*_free_ = 21.7% (Table 2).

**TABLE 1:** Data collection statistics. Values of the highest resolution shell are shown in parentheses.

Wavelength (Å)	1.033
Temperature (K)	100
Space group	P 2_1_ 2_1_ 2_1_
*a*, *b*, *c* (Å)	62.54, 88.58, 97.13
*α*, *β*, *γ* (°)	90, 90, 90
Resolution range (Å)	65.45–1.69 (1.78–1.69)
Total No. reflections	777 158 (116845)
No. of unique reflections	61 204 (8827)
Mean *I*/*σ*(*I*)	14.3 (2.8)
CC_1/2_	0.99 (0.837)
Completeness (%)	100.0 (100.0)
Multiplicity	12.7 (13.2)
*R*_pim_	0.039 (0.433)
*R*_meas_	0.139 (1.592)
*R*_merge_	0.133 (1.531)

**TABLE 2:** Refinement statistics. Values of the highest resolution shell are shown in parentheses.

PDB ID	8QZY
Resolution range (Å)	52.58–1.69 (1.75–1.69)
Completeness (%)	99.97 (99.98)
No. of reflections	61 121 (6030)
*R*_work_	0.1773 (0.2502)
*R*_free_	0.2172 (0.2695)
No. of non‐hydrogen atoms	4634
Protein	4243
Water	391
Average B‐factors	33
Protein	31
Water	37
RMSD	
Bond lengths (Å)	0.012
Bond angles (°)	1.143
Ramachandran plot	
Favored (%)	97.44
Allowed (%)	2.38
Outlier (%)	0.18

The two monomers in the asymmetric unit form a homodimer that is similar to the dimers of most related enzymes by interacting mainly via an antiparallel interaction of the N‐ and C‐terminal β‐strands (Figure 3, center). The interface area between both monomers comprises 740 Å^2^, corresponding to approximately 6% of each monomer's surface (analysis with PISA [50]). The C‐terminal β‐strands engage in an antiparallel sheet‐like interaction, which establishes hydrogen bonds between Y270, E271 and Q273 of both chains, whereas other contacts are of non‐polar nature.

**FIGURE 3:**
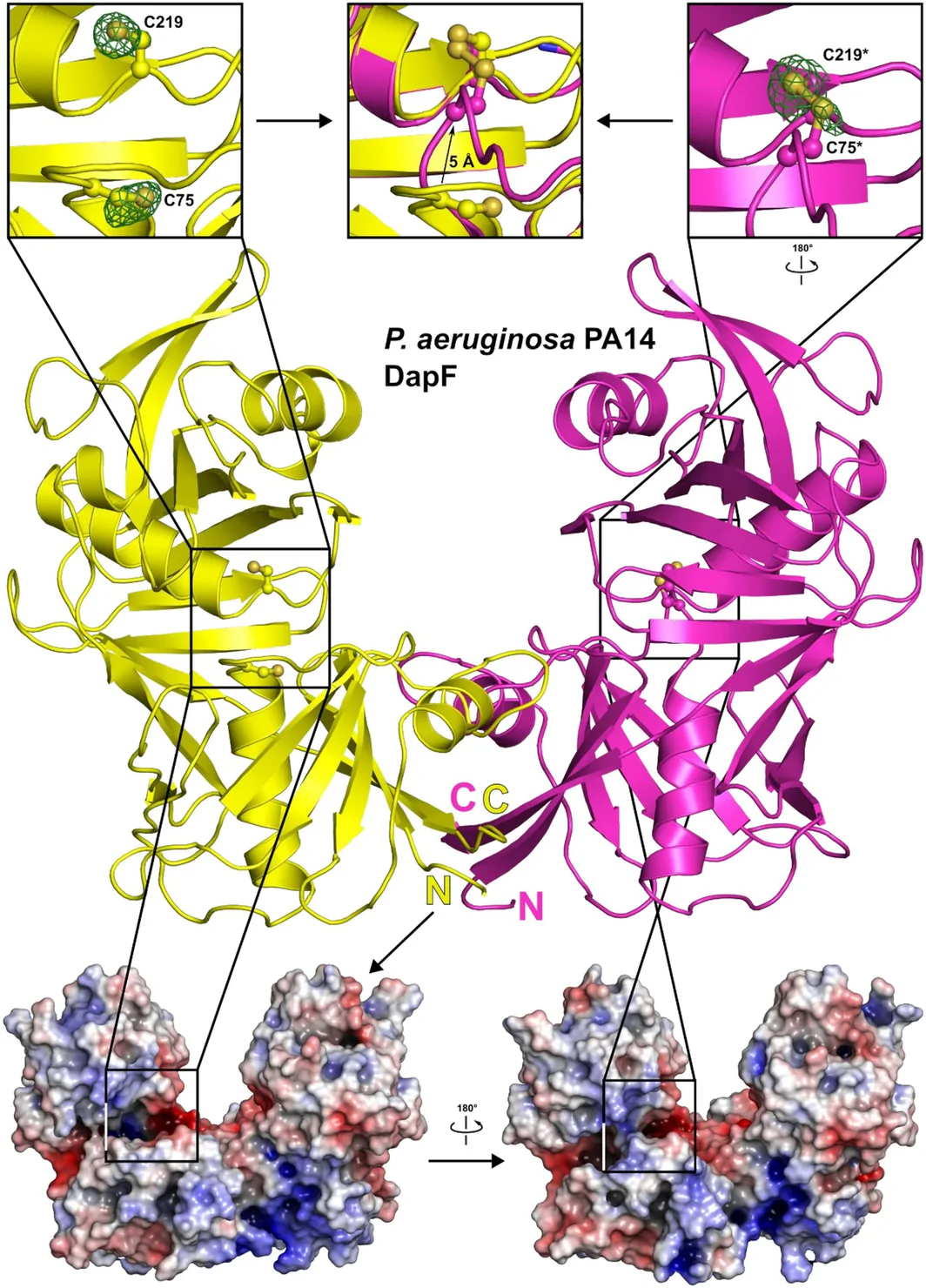
Crystal structure of PA14 DapF. The overall structure of the dimer is shown as a cartoon in the middle of the figure, with active site cysteine residues depicted as ball‐and‐sticks and the position of N‐ and C‐termini indicated. The top row shows 3*σ* |F_O_‐F_C_| electron density around the active site cysteine residues (left and right vignettes). This electron density was generated by removing the side chains of C75 and C219, randomization of the remaining coordinates by 0.5 Å in all directions to remove model bias, followed by re‐refinement in phenix.refine. The central vignette in the upper row shows a superimposition of the two active centers of the DapF dimer to demonstrate the movement of C75 and partial obstruction upon disulfide formation in one of the monomers. The surface potential of the DapF dimer as calculated with the APBS [49] plugin in PyMol [45], displayed at ±5 kT/e with the black rectangle showing the position of the active center, is shown in the bottom row. Note that one active center is in a fully open conformation whereas the second is partially obstructed due to disulfide formation between C75 and C219.

The DapF monomer itself consists of an N‐ and a C‐terminal domain. These domains are structurally very similar and contain a central α‐helix surrounded by eight β‐strands. The N‐termini of the two central α‐helices point at each other at an obtuse angle to contribute their positive dipole moments to the active site (Figure 3, center). In this manner, the *P. aeruginosa* DapF dimer contains two independent active sites that utilize the two cysteine residues C75 and C219 as an acid/base pair to catalyze the epimerization of L,L‐DAP as shown in Figure 1B [16, 30, 51, 52]. It is worth noting that in the analyzed crystal form, the two catalytic cysteines of monomer A are found in the reduced form, whereas they form a disulfide in monomer B. In the oxidized state, C75 and its hosting loop move approximately 5 Å towards C219 with respect to the reduced form, thereby blocking and inactivating the active site without closing it (Figure 3, top and bottom rows). Overall, these observations for PA14 DapF are in good agreement with previously reported DapF structures [16, 21, 22, 31, 32, 51, 52, 53, 54].

Cocrystallization and soaking experiments of *P. aeruginosa* PA14 DapF with L,L‐ or D,L‐DAP did not yield diffracting crystals, despite the fact that temperature sensitivity analysis suggested that these compounds bind the enzyme tightly (see Section 3.1). It is likely that impaired crystallization is rooted in the fact that ligand binding induces significant structural rearrangements to a closed conformation in this family of proteins, hallmarked by an inward movement of the C‐terminal domains that leads to a shielding of the active site (Figure S1) [16, 21, 52]. Because this closing movement is blocked by disulfide formation between C75 and C219 in the second chain, the DapF structure elucidated here shows a state with one open‐active chain (monomer A), whereas the second represents an open‐inactive conformation with very similar relative positions of the N‐ and C‐terminal domains compared to the other chain (Figure 3). The crystal structure of *P. aeruginosa* DapF confirms that both open active and inactive states can co‐exist in a DapF homodimer, however, the importance of this observation that is for allosteric cross‐talk between both active sites or for regulation of the overall enzyme activity, is unclear at present. A similar half‐oxidized form has previously been described for *C. glutamicum* DapF (PDB entry 5H2G), and the authors speculated about a redox mechanism that may regulate the activity of the enzyme [16]. In this context, it is interesting to note that the genome of the cyanobacterium *Anabaena* sp. YBS01 encodes two DapF enzymes, one containing the well‐known cysteine/cysteine and the other a serine/serine active center, which may provide a means to cope with oxidative stress [23]. The physiological relevance of these findings still awaits validation.

### 3.3 Interaction With RppH

Interestingly, in *Escherichia coli* , DapF was found to possess a moonlighting activity that enhances mRNA degradation by interacting with the pyrophosphohydrolase RppH, and it has been speculated that this establishes a link to the metabolism of amino acids [24]. Structural analysis showed that the interaction with RppH is mediated by the N‐terminal domain of DapF, leading to a 2:2 complex with the otherwise monomeric RppH [25, 26]. Complex formation seems to fix RppH in a conformation that favors substrate binding and may also involve interactions between DapF and the RNA substrate [25, 26]. The diaminopimelate activity of DapF, on the other hand, was not required for [24, 25], nor was it altered in the complex with RppH [26], likely because the interaction does not impact on the C‐terminal domain and hence does not impair the formation of a closed conformation in DapF.

Analysis with the AlphaFold3 online server [55] yields a high‐confidence model suggesting that *P. aeruginosa* PA14 DapF and RppH could form a similar complex (Figures 4A and S2A), implicating that DapF may also play a role in mRNA homeostasis in *P. aeruginosa* . We therefore set out to verify complex formation of the *P. aeruginosa* orthologous enzymes experimentally. Towards this, we co‐expressed an His_6_‐tagged *P. aeruginosa* DapF with a Strep‐tagII‐modified variant of *P. aeruginosa* RppH (NudH, YgdP, PA14_04390) and isolated the complex by Strep‐Tactin affinity capture and a subsequent size exclusion step. Interestingly, *P. aeruginosa* RppH was insoluble when expressed without DapF (Figure 4B, rightmost), suggesting that the epimerase's moonlighting function may lie in an RppH‐specific chaperone activity, in a similar manner as we had previously observed for the thioesterase PqsE/quorum sensing transcription factor RhlR couple [56]. SEC‐MALS analysis reveals a molecular weight of 95.9 kDa, which is in between a 2:1 and a 2:2 DapF/RppH complex of the recombinantly expressed affinity‐tagged *P. aeruginosa* enzymes (Figure 4C; expected molecular weight for a 2:2 complex: 107.4 kDa, for a 2:1 complex: 86.1 kDa). This sub‐stoichiometric occupancy of the two RppH binding sites could be a consequence of insufficient resolution of 2:1‐ and 2:2‐complexes in the SEC system. Alternatively, it could result from a rapid equilibrium between a 2:1 + RppH and a 2:2 complex that may lead to a decomposition of the complex via RppH precipitation over time. Similar to the complex in *E. coli* [26], the enzymatic activity of DapF was found unaltered by complex formation with RppH Figure S2B; *K*_*M*_ = 234 μM (95% CI: 210–261 μM) and *V*_max_ = 0.312 μM s^−1^ (95% CI: 0.297–0.328 μM s^−1^), corresponding to a *k*_cat_ of 78 s^−1^ (95% CI: 74–82 s^−1^).

**FIGURE 4:**
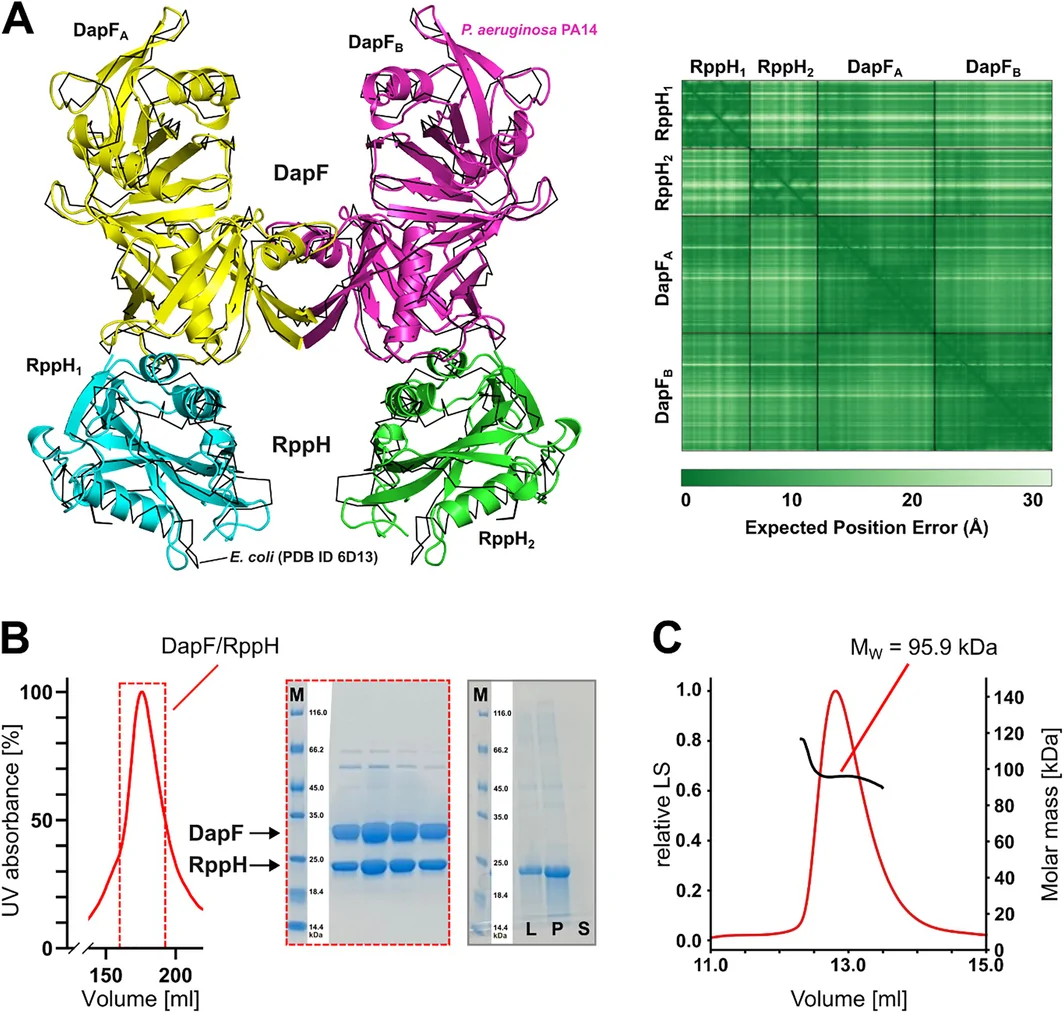
Interaction of DapF with RppH is preserved in *P. aeruginosa* . (A) AlphaFold3 model of a complex between *P. aeruginosa* PA14 DapF and RppH. The crystal structure of the respective *E. coli* complex (PDB code 6D13) is shown in thin black lines. The square on the right shows the expected relative position error as predicted by the AlphaFold3 server [55]. An alternative representation of the predicted complex, colored by pLDDT values, is shown in Figure S2, sequence alignments and contact analysis are shown in Figure S4. (B) Size exclusion chromatography purification of the *P. aeruginosa* PA14 DapF/RppH complex (red), together with the SDS‐PAGE analysis of the indicated region of the elution peak. The SDS gel on the right shows an attempt to overexpress *P. aeruginosa* PA14 RppH in *E. coli* alone (in the absence of *P. aeruginosa* PA14 DapF), leading to insoluble production of the hydrolase (L: Whole‐cell lysate, P: Pellet, S: Soluble fraction). The molecular weight of the respective marker (M) bands in kDa is indicated. The original gels are shown in Figure S4. (C) SEC‐MALS analysis of the *P. aeruginosa* PA14 DapF/RppH complex immediately after Strep‐Tactin affinity purification. The *x*‐axis shows the retention volume of the SEC column. Relative light scattering is shown by the red curve. The black line refers to the right *y*‐axis and shows the calculated molecular weight. Note that because both proteins still contain affinity tags, the expected molecular weight of a 2:2 complex is 107.4 kDa, whereas it would be 86.1 kDa for a 2:1 complex.

## 4 Conclusion

Our data show that the gene PA14_69690 in *P. aeruginosa* PA14 encodes a DapF diaminopimelate epimerase as was expected from sequence similarity and genomic neighborhood. The enzyme is a homodimer and shares many similarities with previously analyzed homologues. Both monomers in the PA14 DapF crystal structure feature a cleft between the N‐ and C‐terminal domains that is typical for the open conformation of this enzyme. In monomer B, however, the catalytic residues are oxidized to a disulfide and are therefore unable to catalyze the epimerase reaction, hence monomer B likely represents an open but inactive state. Since the open active site provides a large cleft that could be used to target this essential enzyme with inhibitors, the two open conformations of PA14 DapF provided here may serve as structural templates for future inhibitor design efforts and help to combat antibiotic resistance of the nosocomial pathogen *P. aeruginosa* . Because modeling and initial experimental validation suggest that PA14 DapF, similar to the *E. coli* protein, could also be involved in RNA homeostasis via its potential interaction with RppH, other avenues to target this enzyme may exist as well.

## Author Contributions

**Philipp Schneider:** writing – original draft, conceptualization, investigation, validation, methodology, formal analysis, data curation. **Stefan Schmelz:** conceptualization, investigation, validation, formal analysis, methodology. **Wulf Blankenfeldt:** conceptualization, investigation, funding acquisition, writing – original draft, writing – review and editing, visualization, project administration, supervision, formal analysis. **Claudia Hanko:** investigation. **Konrad Büssow:** conceptualization, investigation, validation, formal analysis, supervision, methodology.

## Funding

The project was funded by the Lower Saxony doctoral program “Drug Discovery and Cheminformatics for New Anti‐Infectives (iCA)” (21‐78904‐63‐5/19).

## Conflicts of Interest

The authors declare no conflicts of interest.

## Supporting information


**Table S1:** Enzyme kinetic parameters of DapF enzymes.
**Figure S1:** Open/close transition of DapF diaminopimelate epimerase, exemplified by crystal structures of apo and ligand‐bound DapF from *H. influenzae*. The homodimer of apo *H. influenzae* DapF (thin black lines, PDB ID 1GQZ6) has been superimposed onto a homodimer of the same protein bound to the irreversible inhibitor LL‐aziridino‐diaminopimelate (LL‐azidap, shown as sticks, protein shown as cyan/teal cartoon; PDB ID 2GKE7). Electrostatic surfaces at the bottom of this figure have been calculated with the APBS8 plugin in PyMol9, displayed at ±5 kT/e. The position of the active center is indicated by a white oval shape. Note that in the open structure of *H. influenza* DapF, the active center is partially obstructed due to disulfide formation of C73 and C217 (corresponding to C75 and C219 in *P. aeruginosa* PA14 DapF).
**Figure S2:** (A) Complex formation of *P. aeruginosa* PA14 DapF with RppH as predicted with AlphaFold3 [10]. The cartoon has been colored according to the per residue predicted Local Distance Difference Test (pLDDT), showing the high‐confidence in the predicted model. The magnified insert shows the predicted contact area between DapF and RppH. (B) Enzyme‐kinetic characterization of *P. aeruginosa* PA14 DapF (blue) and the DapF/RppH complex (red).
**Figure S3:** Sequence alignments of DapF and RppH from *P. aeruginosa* PA14 and *E. coli*. Secondary structure elements of the respective proteins as found in Figure 4 are shown at the top and bottom, respectively. Identical residues are highlighted in red, contact areas between both proteins in the DapF/RppH complex are highlighted with yellow boxes. The figure has been prepared with ESPript [11].
**Figure S4:** Original gels used for the preparation of Figure 4 in the main manuscript. Boxes indicate the section shown in Figure 4B.

## Data Availability

Atomic coordinates and structure factor amplitudes have been deposited at the Protein Data Bank [46] under accession ID 8QZY.
