# Supplementary material for: Structural and Biochemical Characterization of the Diaminopimelate Epimerase DapF From Pseudomonas aeruginosa PA14
Source: Proteins. 2026 Jul 28;94(12):2002–12. doi: 10.1002/prot.70161 (PMC13728532; doi:10.1002/prot.70161)
Supplement: Supplementary file 1 [file PROT-94-2002-s001.pdf]

Supporting materials

Structural and biochemical characterization of the diaminopimelate epimerase DapF from *Pseudomonas aeruginosa* PA14

Philipp Schneider,[a] Claudia Hanko,[a] Konrad Büssow,[a] Stefan Schmelz[a] and Wulf Blankenfeldt[a,b]

[a]Department Structure and Function of Proteins, Helmholtz Centre for Infection Research, Inhoffenstr. 7, 38124 Braunschweig, Germany

[b]Institute for Biochemistry, Biotechnology and Bioinformatics, Technische Universität Braunschweig, Spielmannstr. 7, 38106 Braunschweig, Germany

Contents:

Table S1 p. 2

Figure S1 p. 3

Figure S2 p. 4

Figure S3 p. 5

Figure S4 p. 6

References for supporting materials p. 7

Tab. S1: Enzyme kinetic parameters of DapF enzymes

**Species**	**K_M (μM)**	**k_{cat} (s^{-1})**
Pseudomonas aeruginosa PA14 (this study)	211	76
Anabaena sp. YBS01, CC-DapF[1]	63	9.5
Anabaena sp. YBS01, SS-DapF[2]	32	2.2
Arabidopsis thaliana[1]	68	3.0
Corynebacterium glutamicum[3]	1860	58
Escherichia coli[4]	124	104
Haemophilus influenzae[5]	700	128

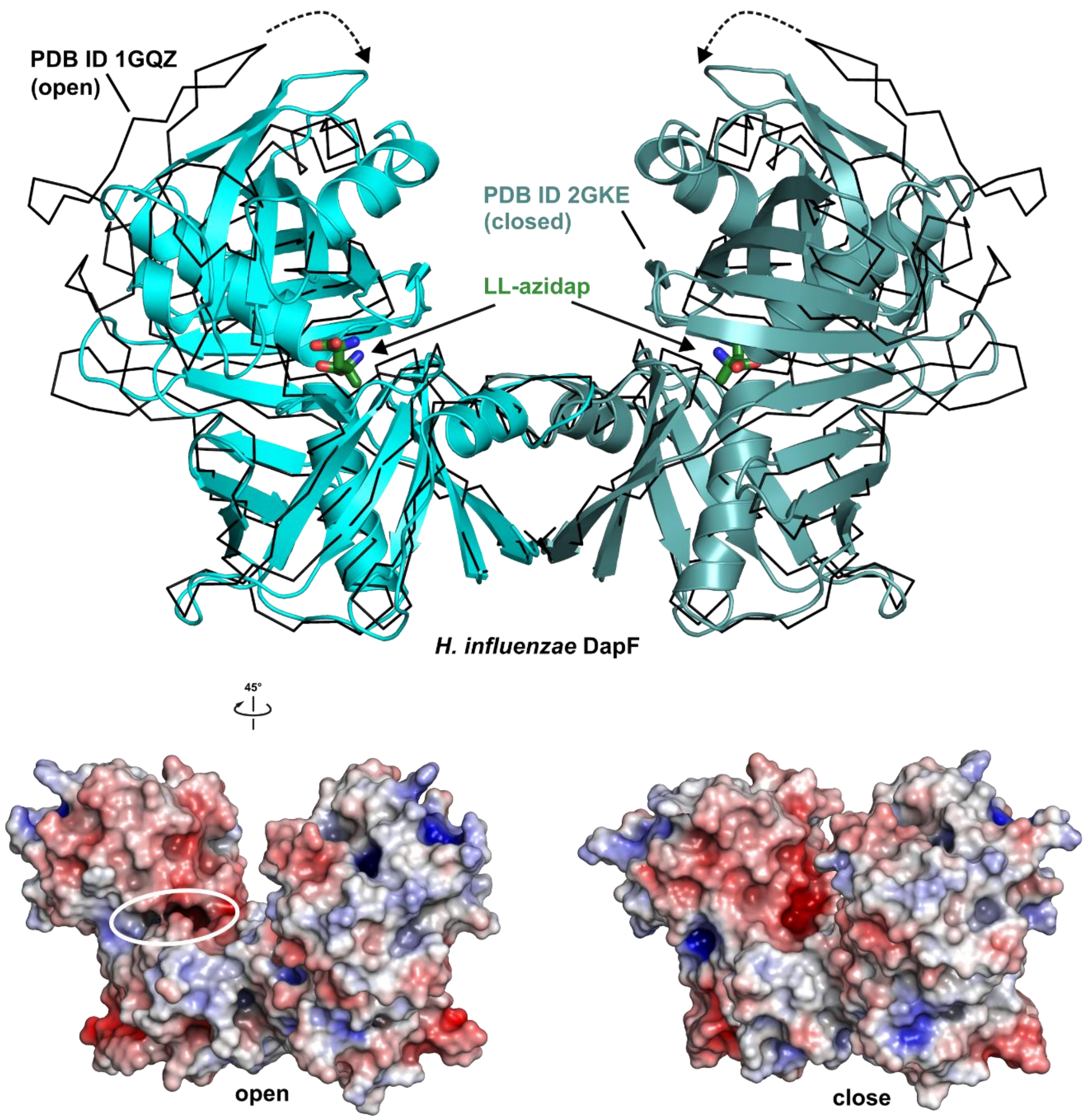

Figure S1 | Open/close transition of DapF diaminopimelate epimerase, exemplified by crystal structures of apo and ligand-bound DapF from *H. influenzae*. The homodimer of apo *H. influenzae* DapF (thin black lines, PDB ID 1GQZ[6]) has been superimposed onto a homodimer of the same protein bound to the irreversible inhibitor LL-aziridino-diaminopimelate (LL-azidap, shown as sticks, protein shown as cyan/teal cartoon; PDB ID 2GKE[7]). Electrostatic surfaces at the bottom of this figure have been calculated with the APBS[8] plugin in PyMol[9], displayed at ± 5 kT/e. The position of the active center is indicated by a white oval shape. Note that in the open structure of *H. influenza* DapF, the active center is partially obstructed due to disulfide formation of C73 and C217 (corresponding to C75 and C219 in *P. aeruginosa* PA14 DapF).

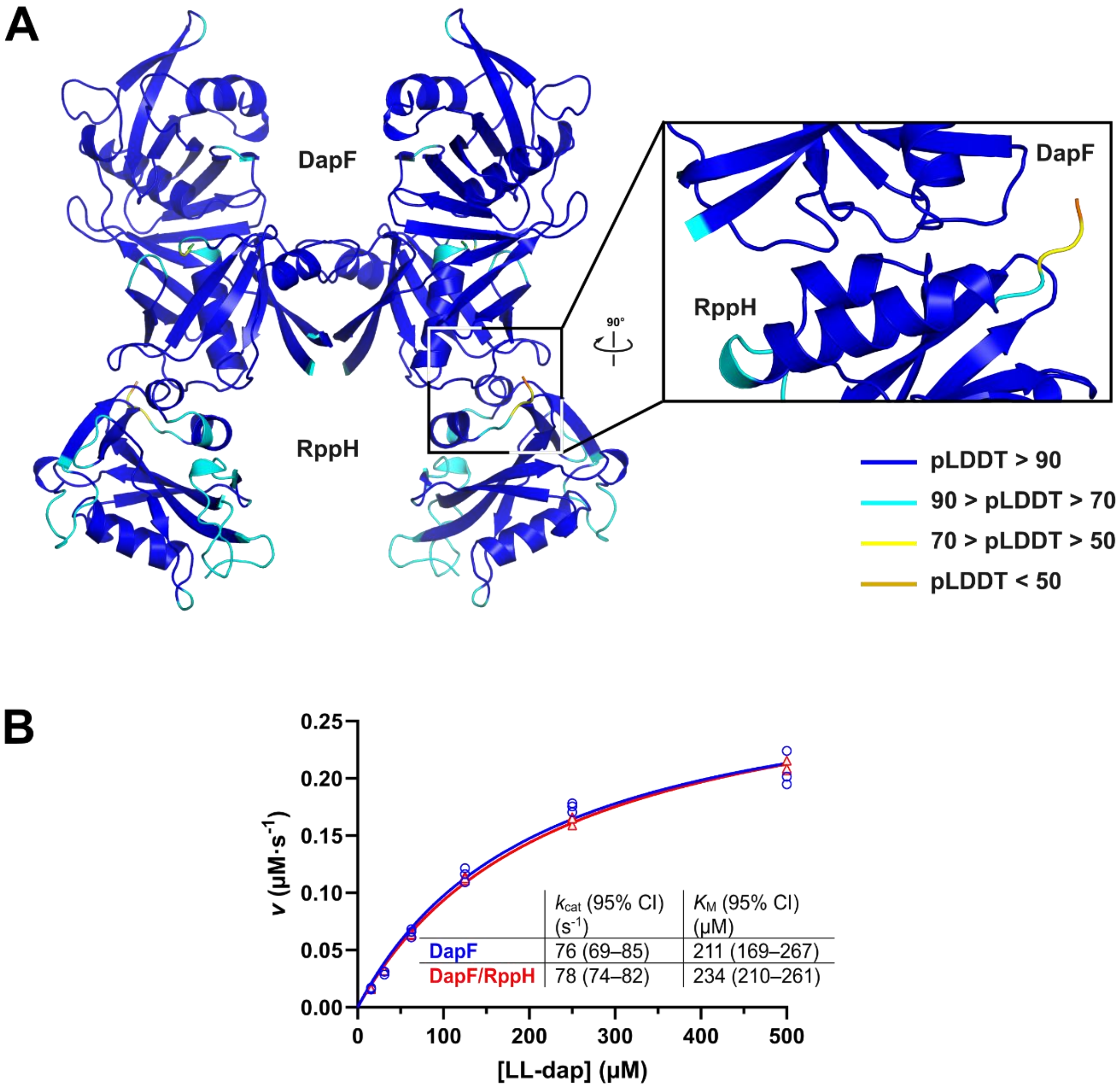

Figure S2 | (A) Complex formation of *P. aeruginosa* PA14 DapF with RppH as predicted with AlphaFold3.[10] The cartoon has been colored according to the per residue predicted Local Distance Difference Test (pLDDT), showing the high confidence in the predicted model. The magnified insert shows the predicted contact area between DapF and RppH. (B) Enzyme-kinetic characterization of *P. aeruginosa* PA14 DapF (blue) and the DapF/RppH complex (red).

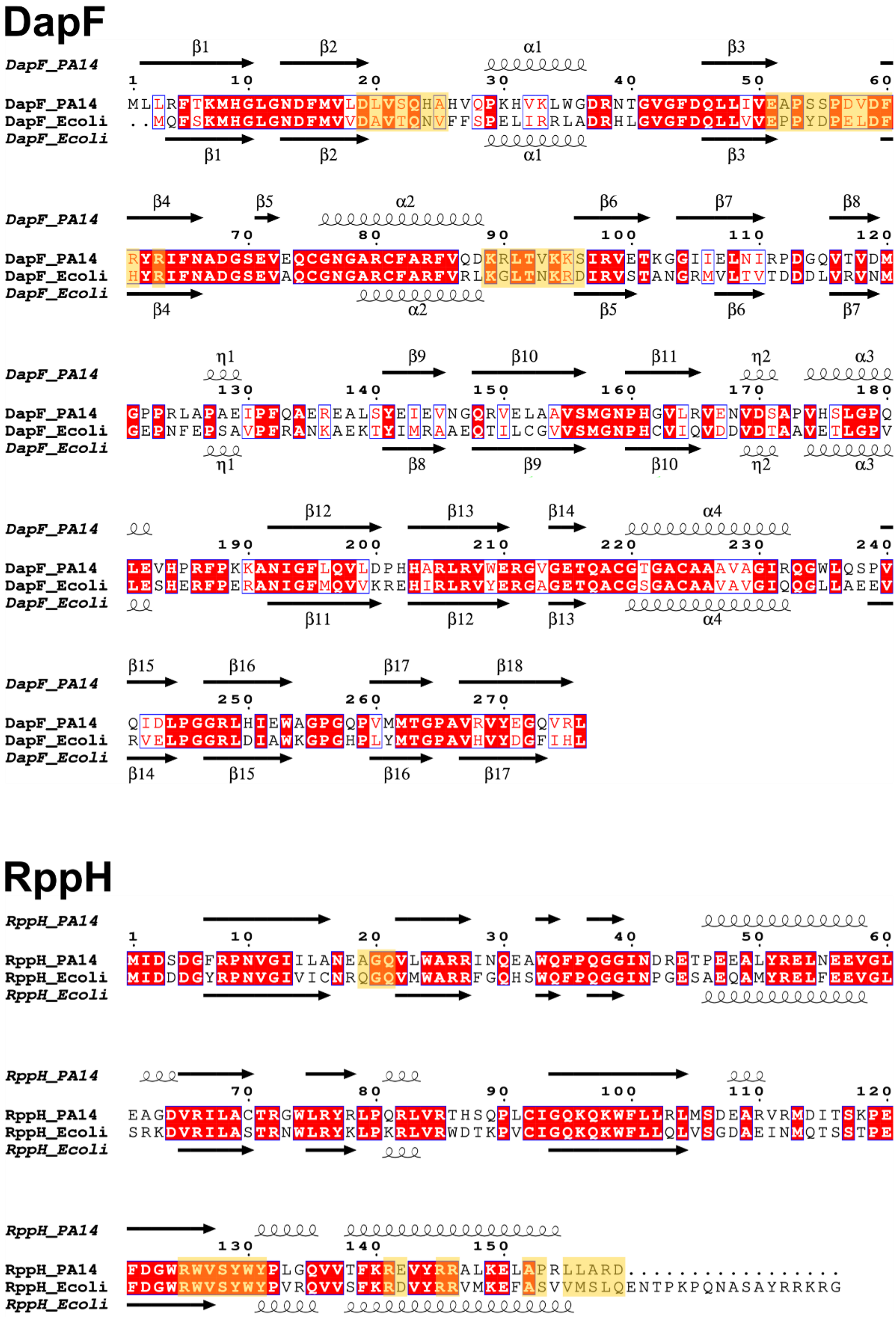

Figure S3 | Sequence alignments of DapF and RppH from *P. aeruginosa* PA14 and *E. coli*. Secondary structure elements of the respective proteins as found in Fig. 4 are shown at the top and bottom, respectively. Identical residues are highlighted in red, contact areas between both proteins in the DapF/RppH complex are highlighted with yellow boxes. The figure has been prepared with ESPript.[11]

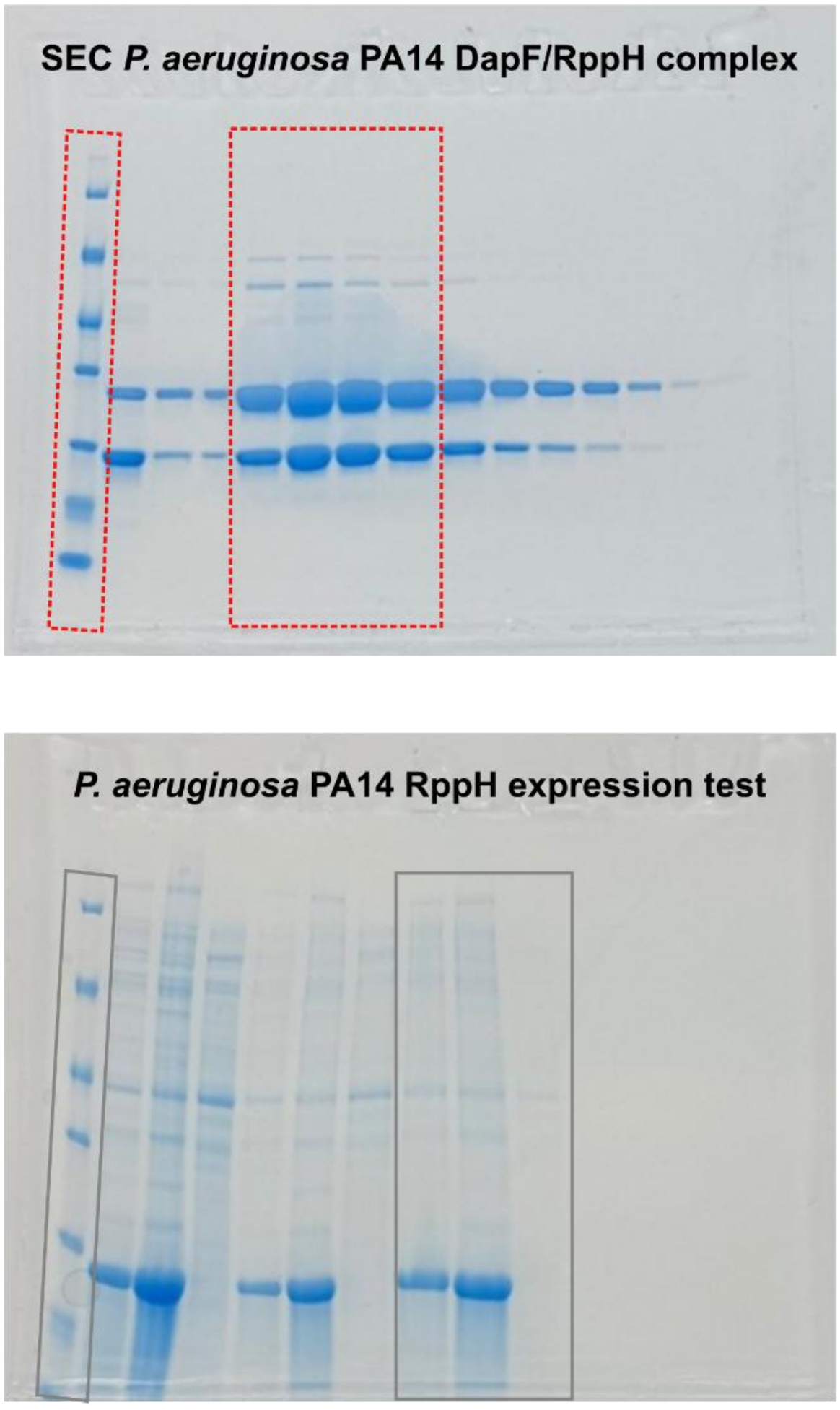

Figure S4 | Original gels used for the preparation of Fig. 4 in the main manuscript. Boxes indicate the sections shown in Fig. 4B.

Supporting references

1. Lamer T, Chen P, Catenza K, et al. Structure and inhibition of diaminopimelic acid epimerase by slow-binding α-methyl amino acids. *Protein Sci*. 2025;34(5):e70139. doi:10.1002/pro.70139

2. Lamer T, van Belkum MJ, Chen P, et al. Cofactor-Independent Amino Acid Epimerases with Catalytic Serines Instead of Cysteines. *J Mol Biol*. 2025;437(21):169375. doi:10.1016/j.jmb.2025.169375

3. Sagong HY, Kim KJ. Structural and functional analysis of DapF. *Scientific Reports*. 2017;7.

4. Hor L, Peverelli MG, Perugini MA, Hutton CA. A new robust kinetic assay for DAP epimerase activity. *Biochimie*. 2013;95(10):1949-1953. doi:10.1016/j.biochi.2013.06.026

5. Koo CW, Blanchard JS. Chemical mechanism of Haemophilus influenzae diaminopimelate epimerase. *Biochemistry*. 1999;38(14):4416-4422. doi:10.1021/bi982911f

6. Lloyd AJ, Huyton T, Turkenburg J, Roper DI. Refinement of Haemophilus influenzae diaminopimelic acid epimerase (DapF) at 1.75 A resolution suggests a mechanism for stereocontrol during catalysis. *Acta Crystallogr D Biol Crystallogr*. 2004;60(Pt 2):397-400. doi:10.1107/S0907444903027999

7. Pillai B, Cherney MM, Diaper CM, et al. Structural insights into stereochemical inversion by diaminopimelate epimerase: an antibacterial drug target. *Proc Natl Acad Sci U S A*. 2006;103(23):8668-8673. doi:10.1073/pnas.0602537103

8. Jurrus E, Engel D, Star K, et al. Improvements to the APBS biomolecular solvation software suite. *Protein Sci*. 2018;27(1):112-128. doi:10.1002/pro.3280

9. Schrödinger, LLC. The PyMOL Molecular Graphics System, Version 3. Published online 2024.

10. Abramson J, Adler J, Dunger J, et al. Accurate structure prediction of biomolecular interactions with AlphaFold 3. *Nature*. 2024;630(8016):493-500. doi:10.1038/s41586-024-07487-w

11. Gouet P, Robert X, Courcelle E. ESPript/ENDscript: Extracting and rendering sequence and 3D information from atomic structures of proteins. *Nucleic Acids Research*. 2003;31(13):3320-3323.
